# Possible Pathophysiological Roles of Neurotransmitter Systems in Men With Lifelong Premature Ejaculation: Protocol for a Scoping Review

**DOI:** 10.2196/41301

**Published:** 2023-03-13

**Authors:** Joost Johan Van Raaij, Paddy Koen Camiel Janssen

**Affiliations:** 1 Department of Clinical Pharmacy and Toxicology VieCuri Medical Centre Venlo Netherlands; 2 Department of Clinical Pharmacy and Toxicology Maastricht University Medical Centre Maastricht Netherlands

**Keywords:** drug therapy, genetic research, lifelong premature ejaculation, scoping review

## Abstract

**Background:**

Lifelong premature ejaculation (LPE) is a rare sexual condition believed to be caused by genetic neurobiological disorders. In the field of LPE, 2 main types of research have been conducted: direct genetic research and pharmacotherapeutic interference of neurotransmitter systems that can relieve the symptoms of LPE in male patients.

**Objective:**

We aim to provide an overview of studies on neurotransmitter systems as the pathophysiological cause of LPE by investigating direct genetic research or pharmacotherapeutic interference that relieves the main symptom of LPE in male patients.

**Methods:**

This scoping review will use the PRISMA-ScR tool (Preferred Reporting Items for Systematic Reviews and Meta-Analyses extension for Scoping Reviews). In addition, this study will use a peer-reviewed search strategy. Systematic searches will be conducted using 5 scientific databases (Cochrane Database of Systematic Reviews, PubMed or MEDLINE, Cumulative Index to Nursing and Allied Health Literature [CINAHL], EMBASE, and Epistemonikos). Additionally, pragmatic searches for relevant information in gray literature databases will be performed. Two reviewers will independently include relevant studies in a 2-stage selection strategy. Finally, data will be extracted from the studies and charted to summarize relevant study characteristics and key findings.

**Results:**

As of July 2022, we completed the preliminary searches according to the PRESS 2015 guidelines and started to determine the final search terms that we will use in all selected 5 scientific databases.

**Conclusions:**

This scoping review protocol is the first to focus on neurotransmitter pathways in LPE by combining the results from the genetic and pharmacotherapy studies. The results could help identify potential research gaps or target candidate proteins and neurotransmitter pathways in LPE for further genetic research.

**Trial Registration:**

Open Science Framework 10.17605/OSF.IO/JUQSD; https://osf.io/juqsd

**International Registered Report Identifier (IRRID):**

PRR1-10.2196/41301

## Introduction

Premature ejaculation (PE) is a common sexual problem among men, with a prevalence of 20%-40% when using a broad definition of the term [1-4]. To obtain valuable standardized and reproducible research on PE, a sharp demarcation of the research field and a clear definition of PE are needed. A distinction can be made between the different types of PE. As defined by a panel of experts at an International Society for Sexual Medicine (ISSM) meeting in 2013, the internationally accepted definition of lifelong premature ejaculation (LPE) and acquired premature ejaculation is as follows [5,6]: (1) Ejaculation that always or nearly always occurs prior to or within about 1 minute of vaginal penetration (LPE) or a clinically significant and bothersome reduction in latency time, often to about 3 minutes or less (acquired premature ejaculation); (2) the inability to delay ejaculation on all or nearly all vaginal penetrations; (3) negative personal consequences, such as distress, bother, frustration, or the avoidance of sexual intimacy.

LPE is a subtype of PE with a prevalence of <4% in the general population [7]. The other suggested subtypes of PE include variable PE and subjective PE. Since these variants are not part of the genetic LPE, they fall outside the scope of this scoping review.

In 1943, Schapiro discovered a possible genetic component in the occurrence of LPE in men [8]. Waldinger further elaborated on this result and thus extended the field of genetic research since 1998 [9]. Thereafter, several studies have examined multiple genes and associated them with the occurrence of LPE [10-27]. Although a clear cause of LPE has not yet been revealed, Schapiro [8] reported that patients responded well to sedatives. Other successful treatments for LPE have been described since 1973 and have been further developed in the past 20 years [28-31]. Some drugs, such as dapoxetine, were specifically registered for the treatment of PE [32]. Additionally, neurobiological causes for LPE are supported by the findings that demonstrate that single-psychotherapeutic treatments or sex therapy is not effective for a significant remission of symptoms [5,33-36]. It has been pointed out that neurotransmitter pathways have multiple complex mechanisms, multiple families, and subtypes of receptors, all of which affect one another, making one polymorphism unlikely to be the sole cause of LPE [10,12,37,38]. It is therefore likely that a subset of polymorphisms in one or more neurotransmitter systems may lead to LPE.

In the field of LPE, 2 main types of research have been conducted. These include genetic research and pharmacological mechanisms that successfully prolong intravaginal ejaculation latency times (IELTs) through pharmacotherapeutic treatment. It is therefore possible to collect evidence on specific neurotransmitter systems. To study neurotransmitter pathways in relation to LPE in this manner, one or more targets can be identified to conduct a targeted strategy for conducting genome-wide association studies (GWAS) and ultimately resolve the cause of LPE.

A preliminary search for the existing scoping and systematic reviews on this topic was conducted on the January 12, 2022, in the following journals, databases, and search platforms: JBI Evidence Synthesis, Cochrane Database of Systematic Reviews, CINAHL (Cumulative Index to Nursing and Allied Health Literature), PubMed or MEDLINE, Embase, and Epistemonikos. Scoping reviews that combined pharmacological evidence with genetic causes were not available.

The objective of this scoping review is to provide an overview of studies on neurotransmitter systems as the pathophysiological cause of LPE by investigating direct genetic research or pharmacotherapeutic interference that relieves the main symptom of LPE in male patients.

## Methods

### Overview

This scoping review will be conducted from April 2022 in 6 stages using the methodology outlined in Levac et al [39], Peters et al [40], and the JBI Manual for Evidence Synthesis [41]. All sections will be reported in accordance with the PRISMA-ScR (Preferred Reporting Items for Systematic Reviews and Meta-Analyses-extension for Scoping Reviews) guidelines [42]. Any amendments will be uploaded on the Open Science Framework public page of this study.

### Identifying the Review Questions

What evidence is currently available that could explain the potentially affected neurotransmitter systems in the pathophysiology of LPE?

The following evidence can be referred: (1) genetic variation correlated with the occurrence of LPE in affected versus unaffected men or (2) successful neuro-targeted pharmacologic treatment of LPE.

### Identifying Relevant Studies

Studies will be collected from the Cochrane Database of Systematic Reviews, PubMed or MEDLINE, CINAHL, Embase, and Epistemonikos databases. Additionally, references of studies will be checked for relevancy and, if eligible, added to this scoping review (snowballing). The reference list of reviews found through the electronic search will be checked to ensure that relevant articles are included in the scoping review. Finally, we will perform pragmatic searches in gray literature databases for relevant information. These include the Grey Literature Report, OpenGrey, Web of Science Conference Proceedings, and local, national, and international organization websites. For inclusion websites should comply with the eEurope 2002 quality criteria [43].

A publication timeline or language restriction will not be set. A systemic search term will be developed using keywords and MeSH terms with the Boolean operators “AND/OR.” Search terms will be applied to the titles and abstracts of the relevant studies. An example of the initial search terms is included in Multimedia Appendix 1. The generated search terms will be peer-reviewed according to the Peer Review of Electronic Search Strategies 2015 guidelines [44]. Google translate will be used as a supportive tool for screening and including non-English studies [45]. Eventually, all included studies will be imported into the Mendeley research software. Studies collected from different sources will be deduplicated in EndNote [46].

### Study Selection

Study selection will occur in 2 stages. The first inclusion will be based on eligibility by screening the title and abstract. Articles that are classified as relevant will be included for full-text review and for final inclusion in the scoping review. The Rayyan software tool will be used for the blind systematic inclusion and exclusion of studies [47]. Studies will be independently selected by 2 individual reviewers. Reviewers will meet when approximately 10%, 50%, and 90% of the studies have been screened to discuss dissent regarding study selection. A PRISMA-ScR flow diagram will be used to describe the study selection process. The diagram will be added to the publication of this scoping review.

All inclusion and exclusion criteria are displayed in Table 1.

**Table 1:** Search strategy using inclusion and exclusion criteria based on the patient population, intervention, comparison, outcomes, and study designs.

Parameter	Inclusion criteria	Exclusion criteria
Patient	Adult males with LPE^a^ (aged ≥18 years)	Populations not matching with one of the following definitions: ISSM^b^ 2007, ISSM 2013, DSM-5^c^, or Definition 1^d^
Intervention	Genotyping or pharmacotherapy treatment	Pharmacotherapy studies using a topical anesthetic or locally active agents or with traditional or herbal medicine
Comparison	Males without LPE in genotype studies or drug naïve or washed-out patients with LPE in pharmacotherapy studies	N/A^e^
Outcome	Comparison of genotype or gene’s or *x*-fold increase in sIELTs^f^ by drug treatment	N/A
Study design	In pharmacotherapy studies: RCTs^g^, cross-over drug studies, single-arm trials with a baseline sIELT, or case reports with a baseline sIELT	N/A

^a^LPE: lifelong premature ejaculation.

^b^ISSM: International Society for Sexual Medicine.

^c^DSM-5: Diagnostic and Statistical Manual of Mental Disorders.

^d^Definition 1: studies using a cut-off IELT of approximately 1.5 minutes after vaginal penetration, measured using the stopwatch method. Symptoms must be present from the first sexual encounter to all sexual partners.

^e^N/A: not applicable.

^f^sIELTs: stopwatch-measured intravaginal ejaculation latency time.

^g^RCT: randomized controlled trial.

### Inclusion Criteria

#### Patient Population

Studies qualify for inclusion if the patient population meets the criteria for LPE, as stated in the ISSM 2007-2013 definition or in the 2013 Diagnostic and Statistical Manual of Mental Disorders (DSM-5) [5,7,48]. IELTs must be measured using the stopwatch method that is the standardized outcome method demanded in drug registration by the European Medicine Association [49]. As these criteria were defined in 2007, the studies performed before this time have a risk of diagnosing patients with LPE with vague terminology or less reliable parameters such as self-reported estimated ejaculatory latency times (eIELTs) using questionnaires [30,50,51]. However, by rejecting the studies executed before 2007, we may overlook useful data; we have therefore designed a secondary inclusion term for the studies performed before 2007. These terms approach the ISSM 2007 or DSM-5 definition as closely as possible. The prior and post-2007 definitions used in the DSM-III [52], DSM IV [53], DSM-IV-TR [54], ICD-9 [55], ICD-10 [56,57], and ICD-11 [58] or the definition used by the American Urological Association Guideline on the pharmacologic management of PE [59] have been found to be inferior to correctly diagnosed LPE [1,60].

Studies before 2007 are eligible if they use a cutoff of approximately 1.5 minutes after vaginal penetration, measured using the stopwatch method. Symptoms must be present from the first sexual encounter to all sexual partners. This additional definition is based on the proposals for definitions published by Waldinger et al in 2006 [1].

#### Pharmacotherapy Studies

We will include the pharmacotherapy studies that meet the following criteria: (1) randomized placebo-controlled trials; (2) cross-over designs; (3) single-arm trials with a baseline stopwatch measuring intravaginal ejaculation latency time (sIELT); and (4) case reports with a baseline sIELT.

### Exclusion Criteria

The exclusion criteria here are pharmacotherapy studies using traditional or herbal medicines and local topical anesthetic agents, as these drugs do not interfere with the central neurotransmission systems.

### Charting the Data

**Figure 1:**
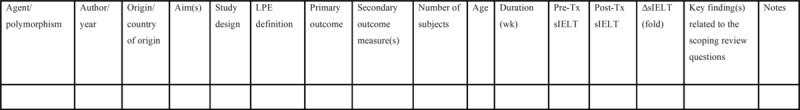
Example of data charting form. LPE: lifelong premature ejaculation; sIELTs: stopwatch-measured intravaginal ejaculation latency time; Tx: drug therapy; wk: week(s).

Data will be collected by the research team with research software that is yet to be specified. Data will be collected as an iterative process, in which reviewers extract and update data continuously. Data will be collected as shown in Figure 1. To test whether the framework matches the research question and aim of the study, 2 reviewers will independently use the data charting form using the data extracted from the first 5 article*s.*

If any data are unclear or incomplete, the corresponding authors will be contacted for additional details on the performed studies. If additional information will not be obtained, a comment on this issue will be placed in the charting table.

### Collating, Summarizing, and Reporting the Results

The key findings of the included studies will be summarized. A distinction will be made between genotype research studies and pharmacotherapeutic drug studies, ordered by the targeted neurotransmission protein subtype. Quantitative data will include, for example, a factor-fold increase in sIELTs by pharmacotherapy or a correlation with specific gene types. This will provide an overview of evidence in the field and could help identify potential research gaps or target protein and neurotransmitter pathways for further genetic research.

## Results

As of July 2022, we completed the preliminary searches according to the PRESS 2015 guidelines and started to determine the final search terms that we will use in all selected 5 scientific databases.

## Discussion

This scoping review protocol is the first to focus on neurotransmitter pathways in LPE by combining the results from the genetic and pharmacotherapy studies. The results could help to identify potential research gaps or target candidate proteins and neurotransmitter pathways in LPE for further genetic research. This could ultimately lead to an improvement in therapy for patients with LPE.

In this scoping review a sharp demarcated definition of LPE will be used to prevent inclusion of non-LPE males by overdiagnosis. Furthermore, the method of this scoping review complies with the most recent guidelines using the PRISMA-ScR tool. In addition, this study will use a peer-reviewed search strategy without language restrictions; therefore, a robust and systematic foundation for thorough scientific research is laid. The final scoping review will be published in a peer-reviewed journal and information will be disseminated in scientific meetings.

This scoping review is limited to wide evidence collection; the quality of evidence of studies will only be partly evaluated since it is not a standard element of scoping review methods. In LPE research the quality of studies is widely discussed [51,61-63]. Confounded published studies are not excluded in advance because this scoping review focusses mainly on wide evidence collection as input for a targeted strategy in a GWAS. To assess potential confounders or outcome differences by ethnicity, patient characteristics and social information of studies will be charted and discussed.

To date several reviews on LPE have been conducted. However, this will be the first scoping review focusing on neurotransmitter pathways in LPE by combining the results from the genetic and pharmacotherapy studies. With this protocol a scoping review could be conducted systemically leading to further input for future candidate gene studies or GWAS and ultimately improve LPE care.

## Appendix

Multimedia Appendix 1 Preliminary search terms.

## Abbreviations

CINAHL: cumulative Index to Nursing & Allied Health Literature DSM-5: Diagnostic and Statistical Manual of Mental Disorders
GWAS: genome-wide association study
IELT: intravaginal ejaculatory latency time
ISSM: International Society of Sexual Medicine
LPE: lifelong premature ejaculation
PE: premature ejaculation
PRISMA-ScR: Preferred Reporting Items for Systematic Reviews and Meta-Analyses-extension for Scoping Reviews
