# Supplementary material for: Possible Pathophysiological Roles of Neurotransmitter Systems in Men With Lifelong Premature Ejaculation: Protocol for a Scoping Review
Source: JMIR Res Protoc. 2023 Mar 13;12:e41301. doi: 10.2196/41301 (PMC10131874; doi:10.2196/41301)
Supplement: Multimedia Appendix 1 [file resprot_v12i1e41301_app1.docx]

**Search terms (preliminary/not final)**

**PubMed 02-05-2022**

| **Search** | **Query** | **Results Pubmed** |
| --- | --- | --- |
| #1 | “Lifelong premature ejaculation”[tw] OR “ejaculatio praecox” [tw] OR lpe OR “rapid ejaculat*”[tw] OR “early ejaculat*”[tw] OR “ejaculatory function” [tw] OR “premature ejaculat*”[tw] OR “anteportal ejaculat*”[tw] OR “primary premature ejaculation” [tw] OR ppe[tw] OR "Premature Ejaculation"[Mesh] | 9,339 |
| #2 | “single nucleotide polymorphism” [tw] OR snp[tw] OR genetic[tw] OR polymorphism*[tw] OR variant*[tw] OR gwas[tw] OR “genome wide association study”[tw] OR genome[tw] OR “genetic varian*”[tw] OR "Genetic Variation"[Mesh] OR "Genome-Wide Association Study"[Mesh] | 2,765,014 |
| #3 | pharmacotherapy[tw] OR “randomized controlled trial*”[tw] OR “randomized controlled trial” [tw] OR “clinical trial” [tw] OR rct[tw] OR “single arm trial” [tw] OR “drug treatment” [tw] OR treatment[tw] OR therapy[tw] OR “drug therapy” [tw] OR “placebo controlled” [tw] OR placebo[tw] OR ielt[tw] OR ielts[tw] OR “intravaginal latency time” [tw] OR “crossover design” [tw] OR “reuptake inhibitor*”[tw] OR "Drug Therapy"[Mesh] OR "Clinical Studies as Topic"[Mesh] | 9,179,719 |
| #4 | mice[tw] OR mouse[tw] OR rat[tw] OR rats[tw] OR animal[tw] OR hamster*[tw] OR “personal protective equipment” [tw] OR “personal protection equipment” [tw] OR “protective personal equipment” [tw] OR “Protective Equipment*”[tw] OR “pooled prevalence estimate” [tw] OR parapneumonic[tw] OR “pleural empyema” [tw] OR “Palmar-plantar erythrodysesthesia” [tw] OR “PPE kit*” [tw] OR “Mycobact*”[tw] OR "Personal Protective Equipment"[Mesh] OR "Tuberculosis"[Mesh] | 4,529,365 |
| #5 | #1 AND #2 | 510 |
| #6 | #1 AND #3 | 3,271 |
| #7 | #5 OR #6 | 3,669 |
| #8 | #7 NOT #4 | 2,206 |
